# CLEC5A mediates Zika virus-induced testicular damage

**DOI:** 10.1186/s12929-023-00906-6

**Published:** 2023-02-17

**Authors:** Hsin-Wei Wang, Hsing-Han Li, Shih-Cheng Wu, Cheng-Kang Tang, Hui-Ying Yu, Ya-Chen Chang, Pei-Shan Sung, Wei-Liang Liu, Matthew P. Su, Guann-Yi Yu, Li-Rung Huang, Chun-Hong Chen, Shie-Liang Hsieh

**Affiliations:** 1 National Institute of Infectious Diseases and Vaccinology, National Health Research Institutes, Zhunan, 350401 Taiwan; 2 National Mosquito-Borne Diseases Control Research Center, National Health Research Institutes, Zhunan, 350401 Taiwan; 3 Division of Biological Sciences, Section of Cell and Developmental Biology, University of California, San Diego, La Jolla, CA 92093 USA; 4 Department of Clinical Laboratory Sciences and Medical Biotechnology, College of Medicine, National Taiwan University, Taipei, 10048 Taiwan; 5 Department of Laboratory Medicine, National Taiwan University Hospital, College of Medicine, National Taiwan University, Taipei, 10021 Taiwan; 6 Program of Plant Protection and Health, Academy of Circular Economy, National Chung Hsing University, Taichung, 402202 Taiwan; 7 Institute of Molecular and Cellular Biology, National Taiwan University, Taipei, 10617 Taiwan; 8 Genomics Research Center, Academia Sinica, Taipei, 11529 Taiwan; 9 Department of Biological Science, Nagoya University, Nagoya, 464-8602 Japan; 10 Institute for Advanced Research, Nagoya University, Nagoya, 464-8601 Japan; 11 Institute of Molecular and Genomic Medicine, National Health Research Institutes, Zhunan, 350401 Taiwan; 12 Immunology Research Center, National Health Research Institutes, Zhunan, Miaoli 35053 Taiwan; 13 Institute of Clinical Medicine, National Yang Ming Chiao Tung University, Taipei, 11221 Taiwan; 14 Department of Medical Research and Education, Taipei Veterans General Hospital, Taipei, 11217 Taiwan; 15 Institute of Immunology, College of Medicine, National Taiwan University, Taipei, 10617 Taiwan

**Keywords:** CLEC5A, Zika virus, Inflammation, Testicular damage, Mouse, Mosquito

## Abstract

**Background:**

Zika virus (ZIKV) infection is clinically known to induce testicular swelling, termed orchitis, and potentially impact male sterility, but the underlying mechanisms remain unclear. Previous reports suggested that C-type lectins play important roles in mediating virus-induced inflammatory reactions and pathogenesis. We thus investigated whether C-type lectins modulate ZIKV-induced testicular damage.

**Methods:**

C-type lectin domain family 5 member A (CLEC5A) knockout mice were generated in a STAT1-deficient immunocompromised background (denoted *clec5a*^*−/−*^*stat1*^*−/−*^) to enable testing of the role played by CLEC5A after ZIKV infection in a mosquito-to-mouse disease model. Following ZIKV infection, mice were subjected to an array of analyses to evaluate testicular damage, including ZIKV infectivity and neutrophil infiltration estimation via quantitative RT-PCR or histology and immunohistochemistry, inflammatory cytokine and testosterone detection, and spermatozoon counting. Furthermore, DNAX-activating proteins for 12 kDa (DAP12) knockout mice (*dap12*^*−/−*^*stat1*^*−/−*^) were generated and used to evaluate ZIKV infectivity, inflammation, and spermatozoa function in order to investigate the potential mechanisms engaged by CLEC5A.

**Results:**

Compared to experiments conducted in ZIKV-infected *stat1*^*−/−*^ mice, infected *clec5a*^*−/−*^*stat1*^*−/−*^ mice showed reductions in testicular ZIKV titer, local inflammation and apoptosis in testis and epididymis, neutrophil invasion, and sperm count and motility. CLEC5A, a myeloid pattern recognition receptor, therefore appears involved in the pathogenesis of ZIKV-induced orchitis and oligospermia. Furthermore, DAP12 expression was found to be decreased in the testis and epididymis tissues of *clec5a*^*−/−*^*stat1*^*−/−*^ mice. As for CLEC5A deficient mice, ZIKV-infected DAP12-deficient mice also showed reductions in testicular ZIKV titer and local inflammation, as well as improved spermatozoa function, as compared to controls. CLEC5A-associated DAP12 signaling appears to in part regulate ZIKV-induced testicular damage.

**Conclusions:**

Our analyses reveal a critical role for CLEC5A in ZIKV-induced proinflammatory responses, as CLEC5A enables leukocytes to infiltrate past the blood-testis barrier and induce testicular and epididymal tissue damage. CLEC5A is thus a potential therapeutic target for the prevention of injuries to male reproductive organs in ZIKV patients.

**Supplementary Information:**

The online version contains supplementary material available at 10.1186/s12929-023-00906-6.

## Introduction

The *Flavivirus* family of viruses, which includes dengue virus (DENV), West Nile virus (WNV), and Japanese encephalitis virus (JEV), has been reported to infect the urogenital system and cause orchitis, an inflammation of the testes [1–3]. Another *Flavivirus*, Zika virus (ZIKV), causes mostly asymptomatic infections, though it has been known to cause mild exanthematous febrile disease (ZIKV fever) and neurotropic manifestations in adults as well as increase the risk of Guillain-Barré syndrome [4]. ZIKV infection in pregnant women is linked to congenital ZIKV syndrome [5], which includes microcephaly and other neurological sequelae [6]. Unfortunately, however, a recent ZIKV outbreak has shown that it, like other *Flaviviruses*, is also able to infect human testicular tissue and cause orchitis [7]. Elucidating the molecular mechanisms of ZIKV-induced pathogenesis is therefore critical for the development of interventions that can prevent sexual-organ tissue damage and preserve fertility in male patients. However, the pathological host factors responsible for ZIKV-induced inflammation and subsequent virus-induced orchitis are unknown.

In infected individuals, ZIKV is detectable in the serum, urine, saliva, and vaginal secretions, and persistent presence in semen has been observed [8]. In fact, although ZIKV is predominantly a vector-borne disease transmitted to humans via *Aedes* mosquitoes during blood feeding [9, 10], transmission via sexual contact in humans has been reported [11]. Of the known flaviviruses, ZIKV affects the male reproductive system the most [12, 13], with viral RNA detectable in the semen of most ZIKV-infected men for 8–12 weeks following disease onset [8, 14–16] and up to a year in some patients [17]. Evidence of this has previously been shown in mice, as ZIKV has been shown to infect testicular tissue explants comprising the testes, epididymal epithelial cells, and urogenital-tract leukocytes in mouse models [18, 19]. Furthermore, ZIKV induces local inflammation and causes orchitis, testicular atrophy, and male infertility in immunosuppressed or immunodeficient mice [4, 20, 21]. ZIKV also causes histological damage in urogenital tracts and testes, resulting in testicular atrophy and infertility in mice [7, 12, 21].

Studies indicates that DV [22] and JEV [23] can induce the phosphorylation of DNAX-activating protein of 12 kDa (DAP12) and the secretion of proinflammatory cytokines via the activation of C-type lectin domain family 5 member A (CLEC5A). CLEC5A is a DAP12-associated Syk-coupled receptor expressed in myeloid cells [24]. Overall, CLEC5A appears to be a promiscuous pattern recognition receptor for flaviviruses, bacteria, and extracellular vesicles from virus-activated platelets. For instance, DV engages CLEC5A to activate NALP3 inflammasomes in inflammatory macrophages [25] and co-activation of CLEC5A and TLR2 by *Listeria monocytogenes* further enhances IL-1 and IL-17 production [26]. Furthermore, platelet-derived extracellular vesicles engage CLEC5A and TLR2 to enhance the formation of neutrophil extracellular trap formation [27]. However, the role of CLEC5A in ZIKV infection has thus far remained obscure.

It is already known that the family of C-type lectins also includes dendritic cell-specific ICAM-3 grabbing nonintegrin (DC-SIGN) and mannose receptor (MR). DC-SIGN is essential for the entry of ZIKV [28] and DV [29] into human dendritic cells, while MR is the primary receptor by which DV enters macrophages [30]. CLEC5A colocalizes with DC-SIGN and MR during viral attachment, and CLEC5A and MR form a heterocomplex to interact with DV in macrophages [31]. While CLEC5A is not involved in viral entry into dendritic cells or macrophages, it is activated by flaviviruses [22, 23] and H5N1 viruses [32] to transduce signaling cascades, thereby inducing the secretion of proinflammatory cytokines, which activates nucleotide-binding oligomerization domain (NOD)-, leucine-rich repeat (LRR)-, and pyrin domain-containing protein 3 (NALP3) inflammasomes [25] and increases osteolytic activity [33]. Recent studies have further demonstrated that DV can activate C-type lectin receptor 2 (CLEC2) in platelets and induce the release of extracellular vesicles to activate CLEC5A in macrophages and neutrophils, leading to proinflammatory cytokine release and the formation of “neutrophil extracellular traps” (NETs) [26, 27]. Furthermore, CLEC5A and other CLECs are critical in virus-induced extracellular vesicle release and NET formation [34]. Combined, these findings suggest that C-type lectins are critical for virus-induced inflammatory reactions and pathogenesis [35], though their role, if any, in ZIKV infection (particularly with regards to the testes) is unknown.

The testes are one of the few immune-privileged regions of the body, since they are protected from immunosurveillance by the blood-testis barrier (BTB), which is maintained via structural and cellular mechanisms like those of the blood–brain barrier (BBB). ZIKV, however, has been shown to infect both human and mouse Sertoli cells (SCs) [12, 36], the somatic cells of the testes, and placental macrophages [37], though the pathological mechanisms remain unclear. We therefore investigated whether CLEC5A, which is highly expressed in monocytes and macrophages, is involved in ZIKV-induced testicular pathology.

Here we report that CLEC5A is involved in the ZIKV-induced testicular inflammatory release of the cytokines tumor necrosis factor alpha (TNF-α) and monocyte chemoattractant protein 1 (MCP-1) as well as the infiltration of Ly6G^+^ neutrophils, all of which result in sperm abnormalities in the semen of ZIKV-infected mice. In addition, CLEC5A disrupts spermatogenesis by affecting testicular germ cells. Combined, this evidence suggests that CLEC5A is responsible for ZIKV-induced testicular injury and is a potential target for preventing ZIKV-induced destruction to the male reproductive organ.

## Methods

### Virus maintenance

ZIKV virus strain PRVABC59 was obtained from the Taiwan Center for Disease Control (provided by Dr. Guann-Yi Yu, NHRI, Taiwan). Virus stocks were propagated in mycoplasma-free Vero cells (also provided by Dr. Guann-Yi Yu), and virus titers were titrated by PFU assay as described previously [55].

### Mosquito maintenance

*Aedes aegypti* (Higgs strain) mosquitoes were fed a 10% sugar solution and raised at 28 °C and 70% humidity. For ZIKV infection, one-week-old female mosquitoes were anesthetized on a cold tray and inoculated with 200 PFU of ZIKV via thoracic injection. Inoculated mosquitoes were maintained under normal rearing conditions for 7 days before conducting the mouse infection experiments.

### Mouse maintenance

WT C57BL/6, *c1ec5a*^*−/−*^ knockout mice, *stat1*^*−/−*^ knockout mice, and *stat1*^*−/−*^*clec5a*^*−/−*^ double-knockout mice (all from the C57BL/6 background) were provided by Dr. Shie-Liang Hsieh (GRC, Academia Sinica, Taiwan). *dap12*^*−/−*^ knockout mice and *stat1*^*−/−*^*dap12*^*−/−*^ double-knockout mice (both on the C57BL/6 background) were ordered from the National Laboratory Animal Center in Taiwan. All experimental mice were males aged 8–12 weeks.

### Mosquito-to-mouse infection experiments

ZIKV-infected mosquitoes were starved for 10 h prior biting WT C57BL/6, *c1ec5a*^*−/−*^, *stat1*^*−/−*^, *stat1*^*−/−*^*clec5a*^*−/−*^, *dap12*^*−/−*^, and *stat1*^*−/−*^*dap12*^*−/−*^ mice. All mice were anesthetized prior to exposure using a mixture of Ketalar (100 mg/kg mouse; Pfizer, Taiwan) and Rompun (16 mg/kg mouse; Bayer Animal Health, Monheim, Germany) administrated intraperitoneally. The mice were then shaved on the ventral side and put in a mosquito-housing cage (10 mosquitoes/cage) for mosquito feeding. Each mouse was bitten by 5–10 mosquitoes. Mouse serum was collected at 2 dpi to test for infectious virus particles and to conduct a serum cytokine expression assay. 9 week old mice (*clec5a*-related experiments) or 12 week old mice (*dap12*-related experiments) were monitored daily for survival, weight loss, and disease symptoms for 7–21 d, depending on the experiment. For hematoxylin and eosin staining, immunohistochemistry, immunofluorescence, and viral titer analysis, the mice were sacrificed, and the testes and epididymis were collected and processed at 7 dpi.

### Infectious virus titer of saliva or whole body in mosquitoes

At 7 dpi, the whole body or saliva collected from 5-day-old female *Aedes aegypti* mosquitoes following thoracic infection with ZIKV (Asian strain PRVABC59 from Taiwan CDC; 200 PFU/mosquito) were examined for ZIKV titer as measured by plaque forming units (PFU) using Vero cells. The saliva was collected into 5% serum-containing DMEM medium and stored at – 80 °C until assayed.

### Histology and immunohistochemistry

Tissues from the ZIKV-infected mice (*stat1*^*−/−*^, *stat1*^*−/−*^*clec5a*^*−/−*^, *dap12*^*−/−*^, and *stat1*^*−/−*^*dap12*^*−/−*^) and uninfected WT mice were collected immediately after perfusion with phosphate-buffered saline (PBS) and fixed overnight in 4% paraformaldehyde. The samples were then stored in 70% ethanol and delivered to the NHRI pathology core lab for paraffin embedding. After deparaffinization, slides were stained with hematoxylin and eosin for histological analyses.

For immunohistochemistry, tissue sections from the ZIKV-infected *stat1*^*−/−*^, *stat1*^*−/−*^*clec5a*^*−/−*^, *dap12*^*−/−*^, and *stat1*^*−/−*^*dap12*^*−/−*^ mice and the noninfected WT mice were boiled in target retrieval solution (K800521, Dako). After the sections had been washed with PBS, 3% H_2_O_2_ was added to quench endogenous peroxidase activity, and PBS containing 10% FBS and 5% BSA was used as a blocking buffer. Tissue sections were incubated overnight at 4 °C with primary antibodies ZIKV NS1 (172–351) antiserum [40], anti-Ly6G (BE0075-1, BioXcell; provided by Dr Li-Rung Huang, NHRI, Taiwan), anti-TRA98 (ab82527, Abcam), anti-CLEC5A, and anti-flavivirus group antigen [D1-4G2-4–15] (AB00230-23.0, Absolute Antibody). The sections were washed with PBS and then incubated with secondary antibodies for 1 h at room temperature. Section staining was performed using a DAB substrate kit (SK-4105, Vector), and the sections were then counterstained with hematoxylin.

### Protein binding assay

To determine the binding ability of CLEC5A to ZIKV, 3 μg of recombinant human CLEC5A protein (Cat: 8544-CL, R&D SYSTEMS) or GFP protein was pre-incubated with ZIKV (5 × 10^6^ PFU) in a reaction volume of 500 μl RIPA buffer at 4 °C for 1 h. The protein-virus mixture was then incubated with 2 μl anti-CLEC5A (Cat No. GTX127349, GeneTex) and a negative control was conducted with 3 μl anti-GFP (Cat No. GTX113617, GeneTex) antibody overnight at 4 °C, followed by immunoprecipitation with 20 µL protein A/G magnetic beads for 3 h at 4 °C. The samples were boiled and resolved by 15% SDS-PAGE. Primary antibodies anti-4G2 (1:2000; Cat: GTX57154, GeneTex), anti-CLEC5A (1:1000), or anti-GFP (1:2000) were used for western blot analysis.

### Detection of DAP12 expression in mouse tissues

Following perfusion with normal saline, tissues from the testis or epididymis of *stat1*^*−/−*^, *stat1*^*−/−*^*clec5a*^*−/−*^ and *stat1*^*−/−*^*dap12*^*−/−*^ male mice with or without ZIKV infection were collected at 7 dpi, homogenized, and prepared for western blot analysis with anti-DAP12 antibody (1:2000; Cat: ab283679, Abcam). Spleen tissues (without saline perfusion) from C57BL/6 mice were used as a positive control for DAP12 expression.

### Sperm examination and ZIKV detection

Mature sperm were isolated from the cauda epididymis as previously described [56], with some modifications. The cauda epididymis was harvested and lysed, then centrifuged for 10 min at 4 °C prior to sperm characterization. The sperm was collected by incubating the harvested tissue in Vitro Fert medium (K-RVFE 50, Cook Medical) for 30 min at 37 °C to allow the sperm to swim out. Sperm suspensions were analyzed for sperm count and sperm motility, conducted within 1 h of the cauda epididymis dissection.

To examine sperm morphology, air-dried sperm smears were prepared, and Papanicolaou staining was performed according to the guidelines of the World Health Organization [57]. In brief, slides were placed in 95% ethanol for at least 15 min for fixation, and then sequentially immersed in 80% ethanol, 50% ethanol, and purified water and were finally stained with Harris’ hematoxylin. After acid ethanol had been used to remove nonspecific staining, slides were sequentially immersed in 50% ethanol, 80% ethanol, and 95% ethanol and were stained with G-6 orange and EA-50 green stains. The slides were dehydrated in 100% ethanol and immersed in xylene for 1 min. Mounting medium (Micromount, Leica) was used to seal the slides.

For ZIKV detection, sperm smears were blocked with PBS (10% FBS, 5% BSA, 0.5% Triton X-100) for 1 h. The sections were incubated overnight at 4 °C with mouse anti-NS1 antibody. Confocal analysis was performed with a TCS SP5 II laser scanning microscope (Leica; Optical Biology Core Lab, NHRI).

### Quantitative real-time polymerase chain reaction (qRT-PCR) analysis

Total RNA was extracted from homogenized mouse tissues using TRI Reagent (Sigma–Aldrich). Complementary DNA (cDNA) was synthesized from 1 μg of RNA using SuperScript II Reverse Transcriptase (Invitrogen) according to the manufacturer’s instructions. Quantitative RT-PCR was performed on a ViiA 7 RT-PCR System (Thermo Fisher Scientific) using a KAPA SYBR FACT qPCR Kit, and gene expression levels were normalized using 18S rRNA. The following primers were used: TNF-α (5′-TCCCAGGTTCTCTTCAAGGGA-3′ forward; 5′-GGTGAGGAGCACGTAGTCGG-3′ reverse), MCP-1 (5′-AGCTGTAGTTTTTGTCACC-3′ forward; 5′-GTGCTGAAGACCTTAGGGC-3′ reverse), CLEC5A (5′-CCAGAAACTGGGATTTTCACCA-3′ forward; 5′-GCCAGTGTGGATCCTTGTGT-3′ reverse), and 18S rRNA (5′-GTAACCCGTTGAACCCCATT-3′ forward; 5′-CCATCCAATCGGTAGTAGCG-3′ reverse).

### Detection of testosterone and cytokines

Serum and testis tissues were harvested from ZIKV-infected *stat1*^*−/−*^, *stat1*^*−/−*^*clec5a*^*−/−*^, *dap12*^*−/−*^, and *stat1*^*−/−*^*dap12*^*−/−*^ mice at 2 or 7 dpi, as well as from uninfected WT mice. Analysis of the serum samples collected at 2 dpi or from uninfected mice was outsourced (SUU-FLOWER, Taiwan), and the concentration of testosterone and cytokines was determined according to standard protocols. Quantitative RT-PCR analysis was used to examine the mRNA expression of TNF and MCP-1 in the testes collected from uninfected mice or at 7 dpi from infected mice.

### Analysis of testicular apoptosis

Testicular tissues from the testes or epididymis of ZIKV-infected (7 dpi) or uninfected mice were removed and then fixed in 4% paraformaldehyde prior to dehydration and paraffin embedding. The 5-µm-thick testis sections were prepared and subjected to TUNEL staining using an In Situ Cell Death Detection Kit (12,156,792,910, Roche) according to the manufacturer’s protocol. The sections were embedded in paraffin and the TUNEL assay was conducted. Briefly, sections were dewaxed and rehydrated in graded concentrations of xylene and ethanol. They were treated with proteinase K working solution for 10 min at room temperature (RT). Detection of TUNEL-positive cells was based on the fixed and permeabilized tissue sections treated with DNase I for 10 min at RT. The sections were washed and then incubated with the TUNEL reaction mixture for 60 min at 37 °C in a humidified atmosphere in the dark. All labeled sections were viewed and imaged using a Leica SP5 confocal microscope.

### Data analysis

Data were analyzed using Prism v6.0 (GraphPad Software). For the immunohistochemistry and immunofluorescence analyses, tissues were collected from at least three mice from each strain. The intensity of staining signals was determined using ImageJ, and the results were analyzed using the Mann–Whitney *U* test. Statistical significance was assumed at *p* < 0.05.

## Results

### CLEC5A is responsible for ZIKV infectivity in testicular tissues

**Fig. 1:**
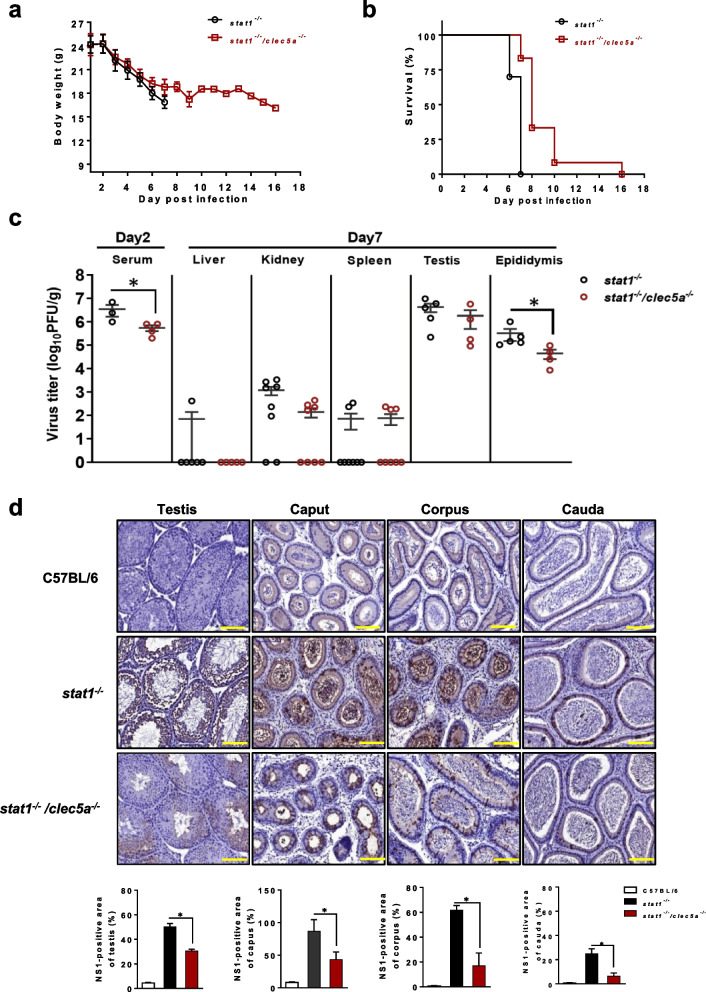
*Clec5a* knockout attenuates ZIKV infectivity in the testicular tissues of mice. Bodyweight (recorded daily) (**a**) and survival (monitored every 2 days) (**b**) in male *stat1*^*−/−*^ and *stat1*^*−/−*^*clec5a*^*−/−*^ mice infected with ZIKV via mosquitoes. **c** ZIKV titer in serum samples (n ≥ 3) collected at 2 days post-infection (dpi) and in various tissue samples (n ≥ 4) collected at 7 dpi. Plaque assay was used to quantify infectious ZIKV and the limit of detection was estimated by counting numbers of plaque formation in original lysates. No plaque formation was observed in the livers, kidneys and spleens of some ZIKV-infected mice. **d** Immunohistochemical staining of tissues from the testes and epididymis (caput, corpus, and cauda) collected from ZIKV-infected mice (n ≥ 4) at 7 dpi and from uninfected C57BL/6 mice (n = 3), which were used as controls. Quantitative data are presented as mean ± standard deviation (SD). *p < 0.05. Scale bars: 100 µm

The immunocompromised STAT1-knockout mouse has emerged as an ideal model for studying ZIKV pathogenesis, as it is highly susceptible to *Flavivirus* infection [22, 23, 27, 38] and lacks active interferon signaling [39, 40]. Conversely, wildtype (WT) C57BL/6 mice are less susceptible to ZIKV infection and do not develop clinically apparent disease [41], resulting in an ideal negative control. To investigate the role of CLEC5A in host pathology during ZIKV infection, we established *clec5a*^*−/−*^ mutant mice, *stat1*^*−/−*^ mutant mice, and *stat1*^*−/−*^*clec5a*^*−/−*^ double-knockout mice. We infected these mice with ZIKV (Asian strain PRVABC59, provided by the Taiwan Centers for Disease Control) via biting by female *Aedes aegypti* mosquitoes carrying ZIKV. The *Aedes* vectors were initially infected with ZIKV (200 plaque-forming units [PFU]) via intrathoracic injection, resulting in a ZIKV titer of approximately 1 × 10^4^ PFU in an entire mosquito or 1 × 10^1^ PFU in the saliva of a mosquito at 7 days post-infection (dpi) (Additional file 1: Fig. S1). Similar to WT mice, ZIKV did not replicate sufficiently in the *clec5a*^*−/−*^ mice to cause any obvious phenotypic differences in body weight and survival (Additional file 1: Fig. S2). Here, the *stat1*^*−/−*^ mice were highly susceptible to ZIKV infection, as indicated by a rapid loss of body weight and early death (< 7 days), while the *stat1*^*−/−*^*clec5a*^*−/−*^ mice were less susceptible but not completely resistant to ZIKV infection, as exhibited by a slower loss of body weight and delayed death (Fig. 1a, b). This suggests that CLEC5A plays a role in the pathogenic effects of ZIKV infection.

We next attempted to home in on the relationship between ZIKV and CLEC5A using a variety of assays. First, because the tissue tropism of ZIKV is closely associated with its pathogenesis, we thus examined the ZIKV titer for various tissues from ZIKV-infected mice. The viral titers in *stat1*^*−/−*^*clec5a*^*−/−*^ mice were slightly lower than those in *stat1*^*−/−*^ mice, but these differences were nonsignificant for all organs except the epididymis (Fig. 1c).

We next assessed whether ZIKV infection of the testicular tissues is affected by the functional presence of CLEC5A which is abundant in testicular tissues (Additional file 1: Fig. S3). As vector- mediated transmission of virus is crucial for initial host infection [42], we investigated potential interactions between the CLEC5A and ZIKV. Using an in vitro immunoprecipitation assay, we found that ZIKA does indeed biochemically interact with CLEC5A (Additional file 1: Fig. S4). Here, we also found that the ZIKV NS1 protein was abundant in the seminiferous tubules of the testes as well as in the caput, corpus, and cauda regions of the epididymis in *stat1*^*−/−*^ mice, which supports previous findings that ZIKV can infect the testes and epididymis [7, 12]. However, there was a decreased viral load in the absence of CLEC5A, as indicated by immunohistochemical analysis. Relative to the levels of NS1 protein in the WT mice, those in the testis and epididymis tissues in both the single-mutant (*stat1*^*−/−*^) and the double-mutant (*stat1*^*−/−*^*clec5a*^*−/−*^) mice were elevated. Importantly, those in the *stat1*^*−/−*^*clec5a*^*−/−*^ mice were lower than those in the *stat1*^*−/−*^ mice (Fig. 1d), suggesting that the loss of CLEC5A attenuates ZIKV infection in the testicular tissues and that CLEC5A may play a role in testicular injury during ZIKV infection.

### CLEC5A contributes to ZIKV-induced orchitis and epididymitis

**Fig. 2:**
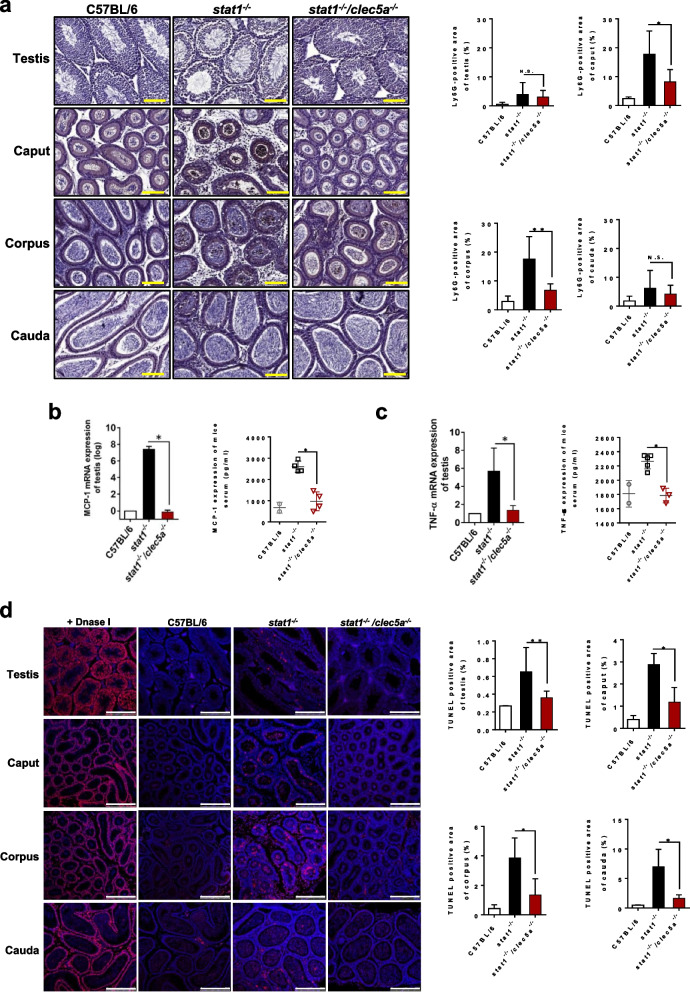
Loss of CLEC5A reduces testicular inflammation and damage. **a** Neutrophil infiltration in the testes and epididymis (caput, corpus, and cauda) collected from ZIKV-infected *stat1*^*−/−*^ mice (n ≥ 8) and *stat1*^*−/−*^*clec5a*^*−/−*^ mice (n ≥ 8) at 7 dpi and from uninfected C57BL/6 control mice (n ≥ 6) was examined and quantified via immunohistochemical staining with anti-Ly6G antibody. Levels of **b** chemokine MCP-1 and **c** proinflammatory cytokine TNF-α in serum and testes from infected mutant mice or uninfected WT mice. The serum was collected at 2 dpi, and the testicular tissues were collected at 7 dpi. The concentrations of MCP-1 and TNF-α in the serum were determined via a standard ELISA assay, and the RNA levels of MCP-1 and TNF-α expressed in the testicular tissues were assessed by quantitative real-time PCR assay. **d** Apoptosis assay using tissue sections from the testes and epididymis (caput, corpus, and cauda) of ZIKV-infected *stat1*^*−/−*^ mice (n ≥ 4) and *stat1*^*−/−*^*clec5a*^*−/−*^ mice (n ≥ 4), with uninfected C57BL/6 mice (n = 3) used as a negative control. DNase I-treated samples served as a positive control. The graphs show the ratio of TUNEL-staining intensity in the ZIKV-infected and uninfected testicular tissue to that in the DNase I-treated samples. Neutrophil invasion and apoptosis in the testicular tissues were evaluated at 7 dpi. Quantitative data are presented as mean ± SD. *p < 0.05; **p < 0.01; N.S., not significant. Scale bars: (**a**) 100 μm; (**d**) 250 μm

ZIKV infection can induce damage in testicular tissues, characterized by orchitis and epididymitis [7]. As CLEC5A was highly expressed in the testicular tissues of *stat1*^*−/−*^ mice (Additional file 1: Fig. S3), we thus investigated whether CLEC5A is involved in ZIKV-induced testicular inflammation using *stat1*^*−/−*^*clec5a*^*−/−*^ double-knockout mice. For this analysis, we stained tissues with anti-Ly6G, a marker for myeloid-derived immune cells such as peripheral neutrophils. We observed massive neutrophil infiltration into the caput and corpus regions of the epididymis in *stat1*^*−/−*^ mice, and found that the loss of CLEC5A compromised leukocyte infiltration in *stat1*^*−/−*^*clec5a*^*−/−*^ mice (Fig. 2a).

To support our observation of leukocyte infiltration associated with CLEC5A, we collected testes samples and serum to examine levels of MCP-1 chemokine, a potent leukocyte attractant. We found that MCP-1 levels in both testes and serum were elevated in *stat1*^*−/−*^ mice infected with ZIKV, but not in the CLEC5A-deficient mutants (Fig. 2b), suggesting that CLEC5A is involved in the infiltration of inflammatory leukocytes into testicular tissues damaged by ZIKV infection. Furthermore, CLEC5A has been proven to play a critical role in flavivirus-induced inflammatory reactions, such as the production and secretion of TNF cytokine [25], but the mechanism underlying how ZIKV induces the production of proinflammatory cytokines remains unknown.

Our data also reveals that CLEC5A facilitates the induction of proinflammatory TNF in testicular tissues upon ZIKV infection, as indicated by the elevated TNF levels in the testes and serum of *stat1*^*−/−*^ mice relative to *stat1*^*−/−*^*clec5a*^*−/−*^ mice (Fig. 2c). In assessing whether the testicular damage caused by ZIKV infection is associated with the presence of CLEC5A, we observed via terminal deoxynucleotidyl transferase dUTP nick-end labeling (TUNEL) assay that testicular apoptosis in *stat1*^*−/−*^ mice was alleviated in the context of CLEC5A deficiency (Fig. 2d). This suggests that ZIKV infection triggers a CLEC5A-involved testicular injury. Together, our data points to a crucial role for CLEC5A in ZIKV-induced testicular pathology.

### CLEC5A is involved in impairing testicular function under ZIKV infection

**Fig. 3:**
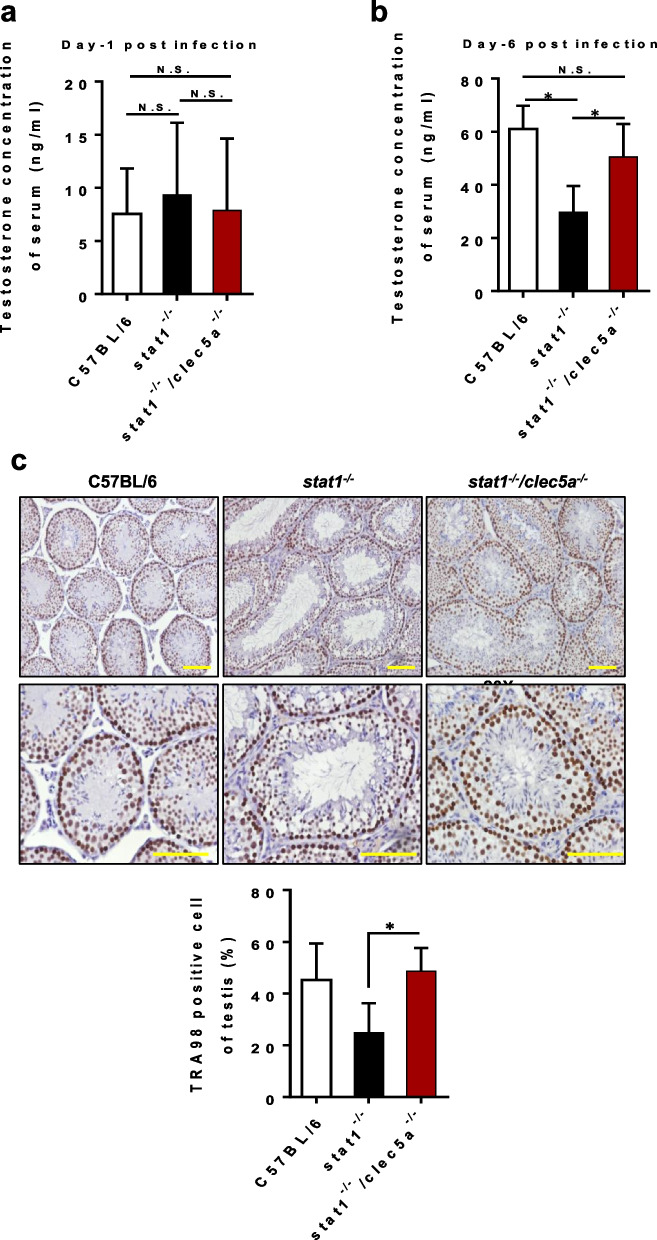
CLEC5A deficiency improves ZIKV-damaged testicular function. Testosterone levels in serum samples from ZIKV-infected mice at 1 dpi (**a**) and 6 dpi (**b**). **c** Immunohistochemical analysis of testicular tissue stained with anti-TRA98. Uninfected C57BL/6 mice (n = 5) served as a negative control for the ZIKV-infected *stat1*^*−/−*^ mice (n = 4) and *stat1*^*−/−*^*clec5a*^*−/−*^ mice (n = 6) at 7 dpi. Quantitative data are presented as mean ± SD. *p < 0.05; N.S., not significant. Scale bars: 100 μm

To investigate whether CLEC5A affects the functionality of ZIKV-infected testes, we assessed whether the level of testosterone, which is important for spermatogenesis, was affected by ZIKV infection. In the early stages of infection (day 1 post infection), there was no obvious difference in testosterone levels between *stat1*^*−/−*^ and *stat1*^*−/−*^*clec5a*^*−/−*^ mice (Fig. 3a). However, testosterone levels in *stat1*^*−/−*^ mice were depressed at 6 dpi. In contrast, testosterone levels were significantly higher in *stat1*^*−/−*^*clec5a*^*−/−*^ mice than in *stat1*^*−/−*^ mice (Fig. 3b), suggesting that CLEC5A may indeed play a role in impairing testicular function.

To confirm this, we analyzed spermatogenesis by quantifying germ cells marked with anti-TRA98, a testis-specific germ-cell marker, via histochemical staining. We observed fewer TRA98-positive cells in the testes of *stat1*^*−/−*^ mice than in WT or *stat1*^*−/−*^*clec5a*^*−/−*^ mice (2.5% vs. 4.4% and 4.9%, respectively; Fig. 3c), suggesting that CLEC5A negatively affects spermatogenesis under ZIKV infection. Depressed sperm production under ZIKV infection has been reported in both humans and mice [7, 43]. Our results indicate that CLEC5A is critical to the ZIKV-induced disruption of spermatogenesis.

### CLEC5A affects ZIKV-induced oligospermia and spermatid malfunction

**Fig. 4:**
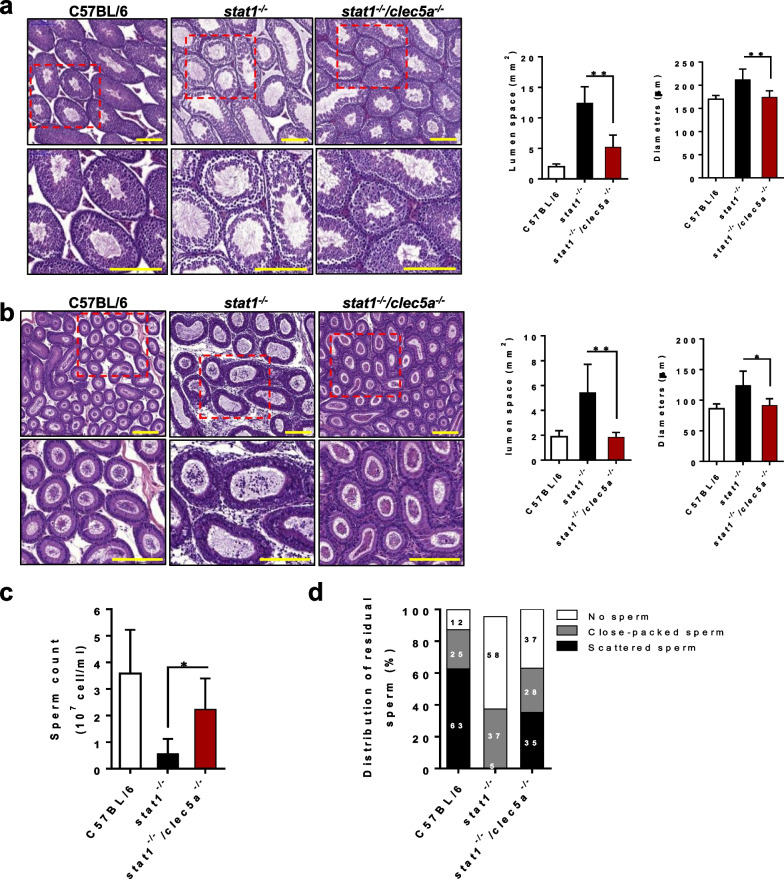
Loss of CLEC5A attenuates oligospermia following ZIKV infection. Hematoxylin-and-eosin staining histological analysis of tissue sections of the seminiferous tubules (**a**) in the testis and (**b**) the mesonephric tubules in the epididymis. **c** Sperm count for epididymis tissues from mice. **d** Spermatid density and distribution in the epididymis of ZIKV-infected *stat1*^*−/−*^ mice (n ≥ 4) and *stat1*^*−/−*^*clec5a*^*−/−*^ mice (n ≥ 5) (7 dpi), with uninfected C57BL/6 mice (n = 6) as negative controls, quantified using Panoramic Viewer software. For a statistical analysis of the distribution of sperm, more than two randomly selected fields of view of the section of the epididymis tissue were assessed for each mouse, and more than three mice were analyzed for each group. Quantitative data are presented as mean ± SD. *p < 0.05; **p < 0.01. Scale bars: 200 μm

ZIKV infection of the testis and epididymis results primarily in a reduction in fertility rate in mice [7, 12], which is strongly correlated with dysfunctional and deficient spermatids. To further investigate the impact of CLEC5A on the quantity and quality of spermatids, we observed the organization and morphology of spermatids in the testes and epididymis of ZIKV-infected *stat1*^*−/−*^ mice. We found fewer and more loosely organized spermatids than in WT mice, and more space in the seminiferous tubules. However, CLEC5A deficiency ameliorated these changes in the double-mutant mice, such that their tissues looked like those of the WT mice (Fig. 4a, b) and the number of spermatids was partially restored (Fig. 4c). In addition, the distribution of spermatids was highly non-uniform in the ZIKV-infected epididymis tissues. Most of the remaining sperm cells in the ZIKV-infected *stat1*^*−/−*^ mouse testes were scattered, and few were closely packed inside the seminiferous tubules (close-packed: 5%; scattered: 37%; none: 58%). In contrast, spermatid distribution in the double-mutant mice (close-packed: 35%; scattered: 28%; none: 37%) was closer to that of the WT mice (close-packed: 83%; scattered: 13%; none: 4%; Fig. 4d). These data suggest that CLEC5A is involved in oligospermia and affects the distribution of residual sperm inside the seminiferous tubules upon ZIKV infection.

**Fig. 5:**
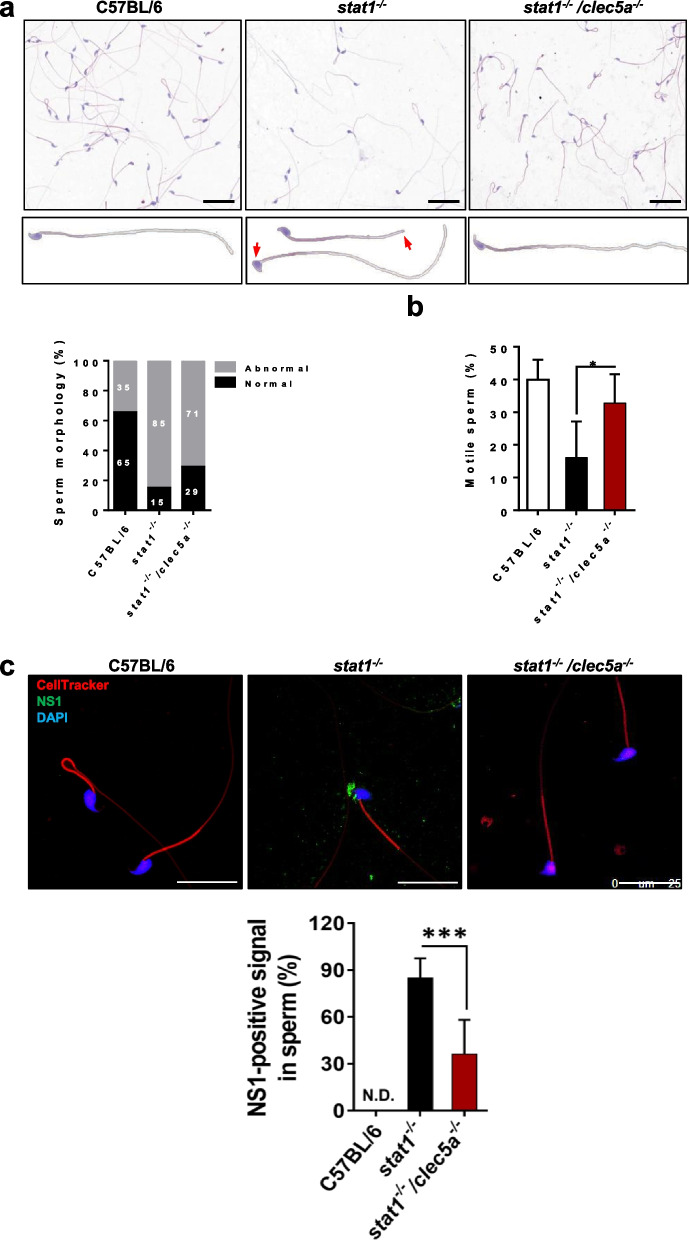
CLEC5A function is related to malfunctioning spermatozoa following ZIKV infection. **a** A cytomorphological study of mature spermatozoa harvested from the cauda of the epididymis was conducted using Papanicolaou stain. Aberrant morphology in the head or tail of spermatozoa (red arrows) was observed in ZIKV-infected *stat1*^*−/−*^ mice and *stat1*^*−/−*^*clec5a*^*−/−*^ mice compared to that in uninfected C57BL/6 mice. The proportions of normal and abnormal sperm were quantified for statistical analysis. **b** Sperm motility assay. At least three randomly selected fields of view were analyzed for each group (**a**). **c** Immunofluorescence staining of sperm allowed the visualization of ZIKV (green, anti-NS1-eGFP), cytoplasm (red, CellTracker), and nuclei (blue, DAPI). ZIKV-positive sperm were counted from more than 150 sperm cells in three random fields of view per mouse and are presented as percentages of all cells. Samples were taken from ZIKV-infected *stat1*^*−/−*^(n = 12) and *stat1*^*−/−*^*clec5a*^*−/−*^ mice (n = 8) (7 dpi) and uninfected C57BL/6 mice (n = 3), which served as negative controls. Quantitative data are presented as mean ± SD. *p < 0.05; ***p < 0.001; N.D., not detected. Scale bars: (**a**) 50 μm; (**c**) 25 μm

We also found that CLEC5A deficiency not only reduced the morphological abnormalities (Fig. 5a) but also elevated the motility (Fig. 5b) of spermatozoa relative to the aberrant morphology and poor motility of the residual spermatozoa in ZIKV-infected *stat1*^*−/−*^ mice. Additionally, infectious ZIKV has been detected in mature sperm from epididymis tissues [12], and the presence of the virus may impair the function of the spermatozoa. Via anti-NS1 staining, we observed a high proportion of NS1-positive spermatozoa (85%) and poor sperm motility in ZIKV-infected *stat1*^*−/−*^ mice. In ZIKV-infected *stat1*^*−/−*^*clec5a*^*−/−*^ mice, we observed a lower proportion of NS1-positive spermatozoa (36%) and higher sperm motility (Fig. 5c), suggesting that CLEC5A plays an important role in increasing ZIKV infectivity in spermatozoa. Combined, these data support the idea that CLEC5A may be a pivotal mediator of testicular damage, and that it may be associated with the oligospermia and spermatid malfunction resulting from ZIKV infection.

### CLEC5A-associated DAP12 signaling is involved in ZIKV-induced testicular damage

**Fig. 6:**
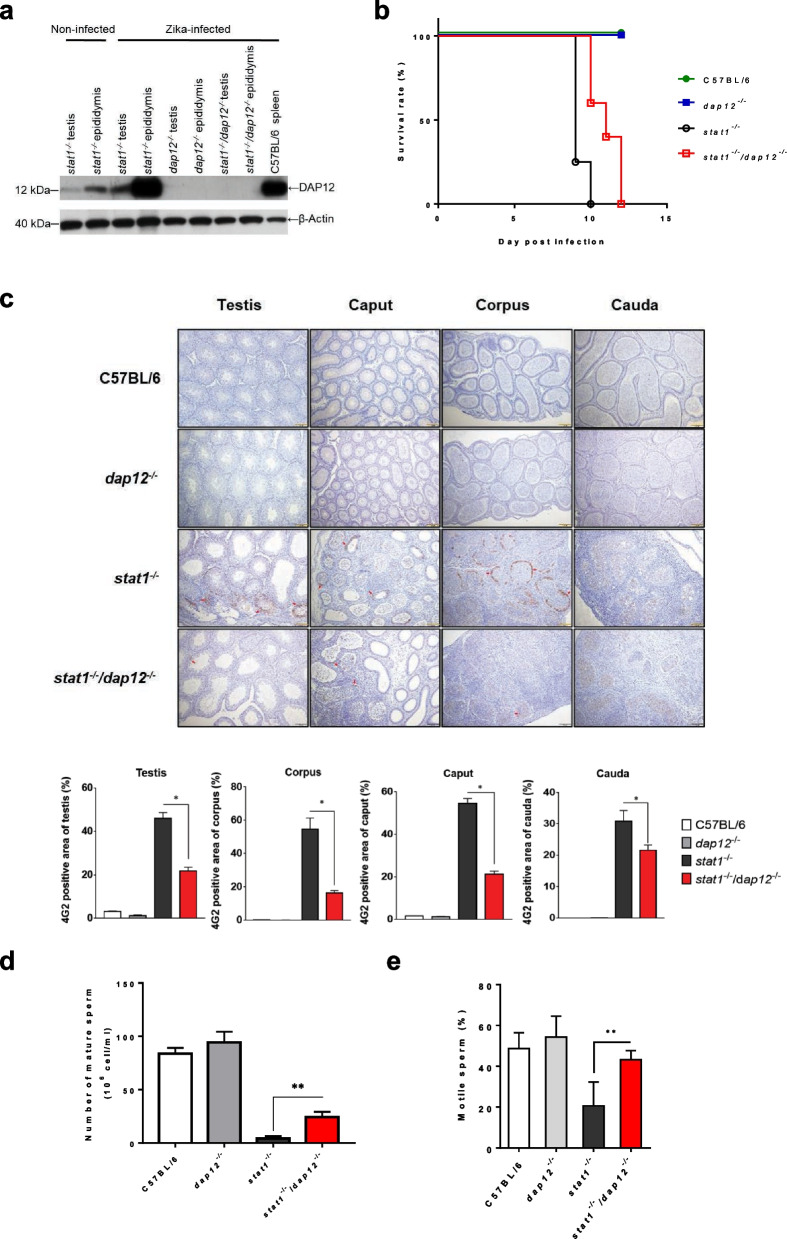
Loss of DAP12 compromises ZIKV infectivity and spermatozoa function in ZIKV-damaged testicular tissues. **a** Western blot analysis of DAP12 protein expressed in various testicular tissues of male *stat1*^−/−^, *dap12*^−/−^, or *stat1*^−/−^*dap12*^−/−^ mice infected with ZIKV at 7 dpi. Spleen tissues with known high DAP12 expression were collected from C57BL/6 mice as a positive control. **b** Survival (monitored daily) in male mutant or C57BL/6 mice infected with ZIKV via mosquito. **c** Immunohistochemical staining (with anti-4G2) of tissues from the testes and epididymis (caput, corpus, and cauda) collected from ZIKV-infected mice (n ≥ 3) at 7 dpi and from uninfected C57BL/6 mice (n = 3) as controls. Scale bars: 100 µm. Quantitative data show the percentage of ZIKV-infected area in different tissues. Sperm count for epididymis tissues (**d**) and sperm motility (**e**) in mutant or C57BL/6 mice with ZIKV infection. For **b**–**e**, *stat1*^−/−^, *dap12*^−/−^, or *stat1*^−/−^*dap12*^−/−^ mutant mice were used. At least three randomly selected fields of view were analyzed for each group. Quantitative data are presented as mean ± SD. *p < 0.05. **p < 0.01

It has been reported that activation of CLEC5A induces the release of proinflammatory cytokines via DAP12 [22, 44], a key accessory protein for relaying signals by the DAP12-associated receptors, such as NK receptors [45] and CLEC5A [22]. As CLEC5A deficiency results in decreased DAP12 expression in testicular tissues (Additional file 1: Fig. S6), we were thus interested to know whether DAP12 contributes to ZIKV-induced testicular damages in *stat1*^*−/−*^*dap12*^*−/−*^ mice. We found that DAP12 is abundantly expressed in the testis and epididymis, and its expression level within testicular tissues was upregulated post-ZIKV infection (Fig. 6a). Compared to *stat1*^*−/−*^ mice, the mortality (Fig. 6b) and ZIKV titer of testicular tissue (Fig. 6c and Additional file 1: Fig. S7) were partially rescued in *stat1*^*−/−*^*dap12*^*−/−*^ mice. Moreover, the levels of proinflammatory cytokines (IL-9, MIP-1a, MIP-1b, Eotaxin, and G-CSF) in *stat1*^*−/−*^*dap12*^*−/−*^ mice were much lower than *stat1*^*−/−*^ mice as determined by ELISA. This demonstrates that DAP12 is involved in ZIKV-induced proinflammatory cytokine release (Additional file 1: Fig. S8).

In contrast, the transcription and translation of TNF and MCP1 in serum and testicular tissue were not significantly altered in *stat1*^*−/−*^*dap12*^*−/−*^ mice post ZIKV infection (Additional file 1: Fig. S9), suggesting that TNF and MCP1 secretion may not be regulated via DAP12. In addition, post-ZIKV infection testicular function in *stat1*^*−/−*^*dap12*^*−/−*^ mice was higher than that of *stat1*^*−/−*^ mice in terms of the number of mature sperm and their motility (Fig. 6d, e and Additional file 1: Fig. S10). These observations suggest that CLEC5A-involved neutrophil chemotaxis, proinflammatory cytokine release (except from TNF and MCP1), and testicular damage is mediated via DAP12.

## Discussion

To improve our knowledge of the mechanisms of ZIKV pathogenesis, we herein investigated the role of CLEC5A in this process. By comparing the responses of ZIKV-infected *stat1*^*−/−*^ and *stat1*^*−/−*^*clec5a*^*−/−*^ mice, we have clearly demonstrated for the first time that CLEC5A deficiency attenuates orchitis, based on the reduction of leukocyte infiltration and levels of the proinflammatory cytokine TNF. In addition, the partial rescue of spermatid counts and motility under ZIKV infection by *clec5a* knockout implicate CLEC5A as a potential target for therapies to treat ZIKV-infection-induced orchitis.

In this study, the absence of CLEC5A suppressed ZIKV-induced inflammatory responses, such as increased expression of TNF and MCP-1 and leukocyte infiltration into the testicular tissues, and thus reduced testicular damage (Fig. 2). This supports the known function of CLEC5A as playing a canonical role in immune–cell-triggered testicular inflammation. We have shown that CLEC5A is not involved in DV entry [22] to macrophages and JEV entry to microglia [23]. Interestingly, the administration of an anti-CLEC5A mAb restored the integrity of the BBB and reduced neuroinflammation and JEV titer in the brain post-JEV infection. Here, the reduced JEV titer is due to the retained integrity of the BBB, which thus reduces the amount of JEV that can access the brain [23].

Here, we demonstrated that CLEC5A was highly expressed in the testicular tissue (Additional file 1: Fig. S3), and a CLEC5A deficiency reduced testicular damage and ZIKV infectivity to the testis (Fig. 1d). This agrees with previous work showing that CLEC5A knockdown decreased the viral load in adult human monocytes post-ZIKV infection [46]. We also demonstrated here that ZIKV interacts with CLEC5A directly, as a CLEC5A deficiency reduced ZIKV entry into the testicular tissues (Fig. 1d). Thus, CLEC5A deficiency may regulate ZIKV entry via (a) reducing the viral load in infiltrating monocytes and (b) maintaining the integrity of the BTB.

CLEC5A has been shown to transduce signals via adaptor DAP12 after activation in myeloid cells [24, 47]. It is interesting to note that a DAP12 deficiency ameliorated ZIKV-induced testicular damage and infectivity but did not affect the secretion of TNF or MCP-1 (Additional file 1: Fig. S8). This observation suggests that the secretion of TNF and MCP-1 may not occur via DAP12. It has been reported that CLEC5A also associated with another adaptor protein DAP10, which is abundant in tissue macrophages [48]. Thus, the secretion of TNF and MCP-1 may occur via DAP10 after CLEC5A activation. Future studies are needed to clarify the role of DAP10 and DAP12 in CLEC5A-involved inflammatory cytokine release and tissue damage after viral infections.

It is interesting to note that lower ZIKV titers were found in the serum, epididymis (Fig. 1c), and brain (Additional file 1: Fig. S5) in CLEC5A-deficient mice. We suspect this is related to the presence of the BBB and the blood-epididymis barrier (BEB) [49, 50], which isolates the brain and epididymis from blood vessels. Previously, we showed that CLEC5A is the pattern recognition receptor for JEV. Moreover, JEV can penetrate the BBB and infect brain macrophage (microglia) and neuronal cells, thus inducing neuronal inflammation and causing BBB damage in WT mice [23]. Compared to WT mice, *clec5a*^*−/−*^ microglia produce far fewer proinflammatory cytokines, and the BBB integrity remains relatively intact. Therefore, less JEV and neuronal deaths are observed in CLEC5A-deficient mice [23]. Similarly, ZIKV can penetrate the BEB to infect Sertoli cells [36] and activate epididymis tissue macrophages [51], thereby causing orchitis and BEB damage in WT mice. Compared to WT mice, *clec5a*^*−/−*^ tissue macrophages produce fewer proinflammatory cytokines, leaving the BTB relatively intact and reducing tissue damage and viral replication in CLEC5A-deficient mice. The relatively intact BTB further limits ZIKV release to the blood stream in CLEC5A-deficient mice (Additional file 1: Fig. S11).

Overall, our results show that CLEC5A deficiency ameliorated the decline of testicular function and sperm count which are clinical features of ZIKV infection [52, 53]. Additionally, the literature shows that the ZIKV infection of pregnant women can also result in congenital neonate malformation, principally as microcephaly caused by interactions between ZIKV and cells in the fetal central nervous system [54]. As multiple viral entry receptor mechanisms underlying this infection of the central nervous system have been proposed [54], we investigated the brain tissue of infected mice, showing that the elevated ZIKV titer in the brain tissue of infected *stat1*^*−/−*^ mice was attenuated by the absence of CLEC5A (Additional file 1: Fig. S5). From our results, we therefore suspect that in addition to its role in testicular injury, CLEC5A might also play a role in ZIKV-induced neurological injury. Further investigations are needed to confirm this role in fetal mouse brains, as infection in the adult model does not have clear relevance to human infections.

## Conclusion

In conclusion, our ZIKV infection mouse model shows for the first time that CLEC5A exacerbates ZIKV infectivity. In mice with normal CLEC5A expression, leukocyte infiltration and the activation of proinflammatory cytokine expression caused damage to testicular tissues, reduced testosterone levels, and malfunctional sperm. This ZIKV-induced testicular pathology was attenuated by CLEC5A deficiency, suggesting that targeting CLEC5A has potential as a therapeutic strategy for improving ZIKV-induced orchitis and associated illness.

## Supplementary Information

**Additional file 1.** Supplementary figures 1–10.

## Data Availability

All data generated or analysed during this study are included in this published article [and its additional files].
