# Supplementary material for: CLEC5A mediates Zika virus-induced testicular damage
Source: J Biomed Sci. 2023 Feb 17;30:12. doi: 10.1186/s12929-023-00906-6 (PMC9936774; doi:10.1186/s12929-023-00906-6)
Supplement: Supplementary file 1 — Additional file 1. Supplementary figures 1–10. [file 12929_2023_906_MOESM1_ESM.pdf]

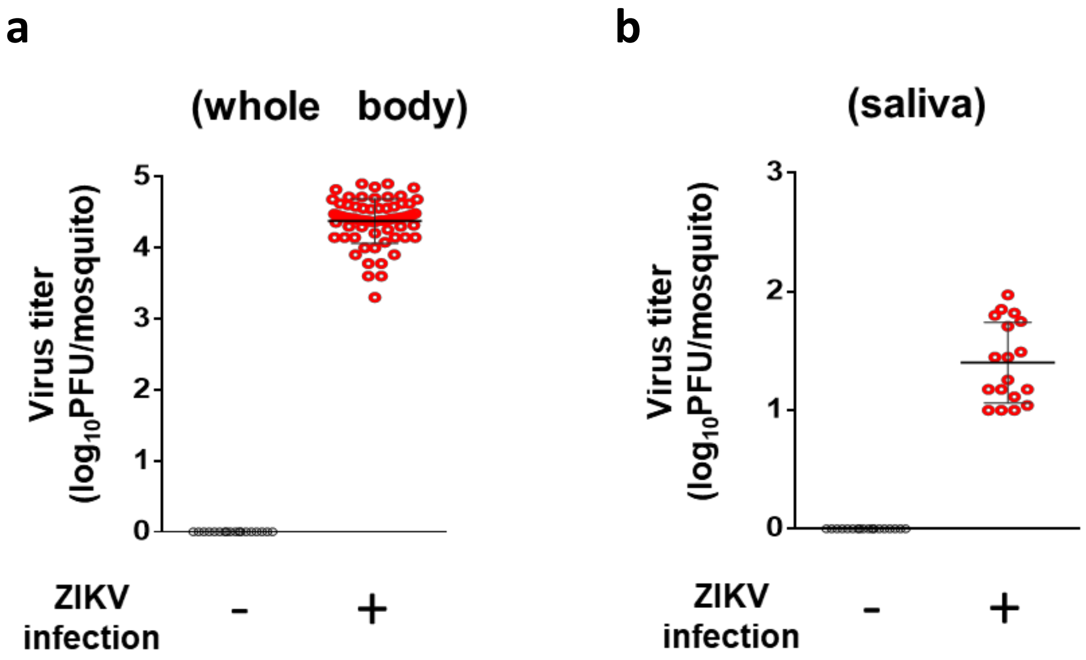

Supplementary Figure 1. ZIKA titer is increased in mosquitoes following ZIKV infection. a,b ZIKA viral titer in whole body (**a**) or saliva (**b**) from ZIKA-infected female *A. aegypti* mosquitoes (n = 70 for a, n = 18 for b) or non-infected female *A. aegypti* mosquitoes (n = 20 for a, b). The viral titers in the mosquito body or saliva were determined by plaque-forming unit assay following thoracic injection with ZIKV at 7 dpi.

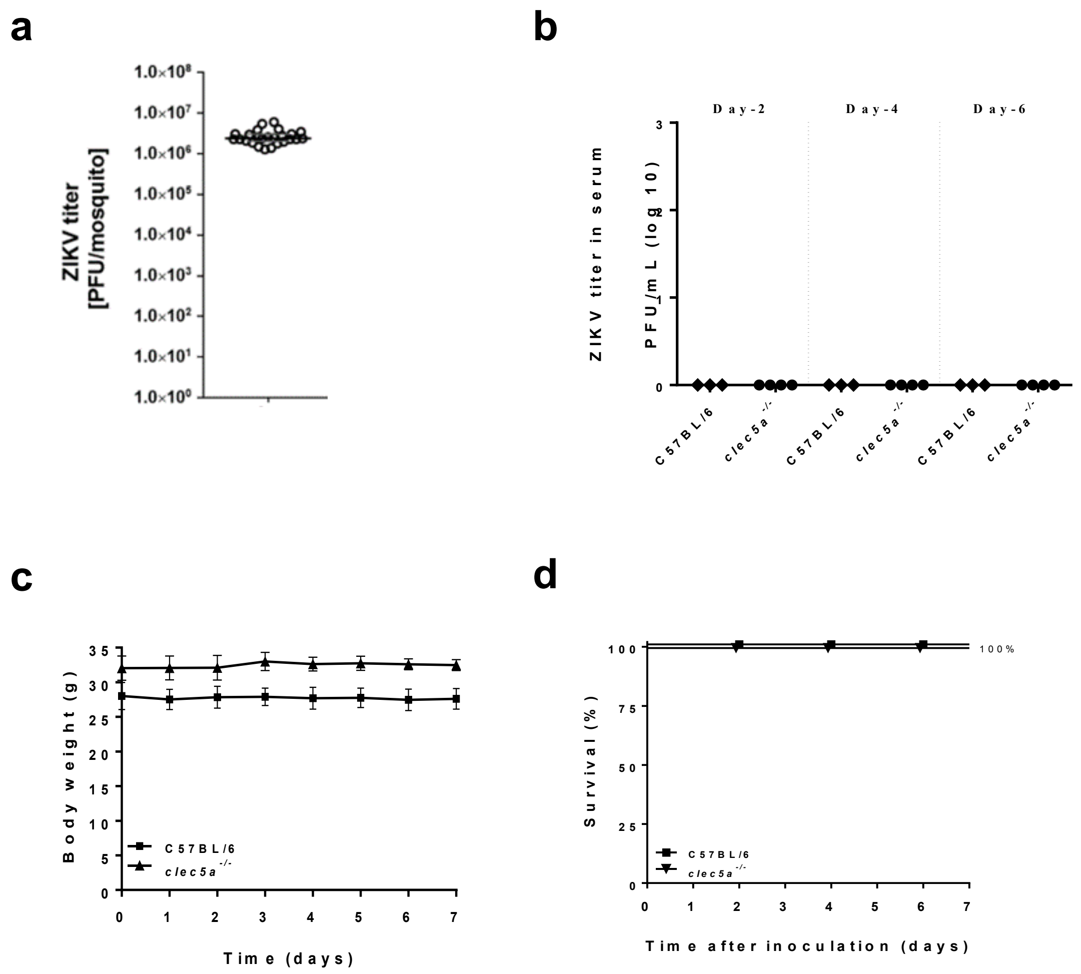

Supplementary Figure 2. **ZIKA causes no obvious detriment to *clec5a$^{-/-}$* mice. a** ZIKA titer in female *A. aegypti* mosquitoes were determined by plaque-forming unit assay following thoracic injection with ZIKV at 7 dpi. **b-d** ZIKV titer in serum (**b**), body weight (**c**), and survival (**d**) in *clec5a$^{-/-}$* female mice (n = 4) compared to control C57BL/6 mice (n = 3) bitten by ZIKV-infected mosquitoes.

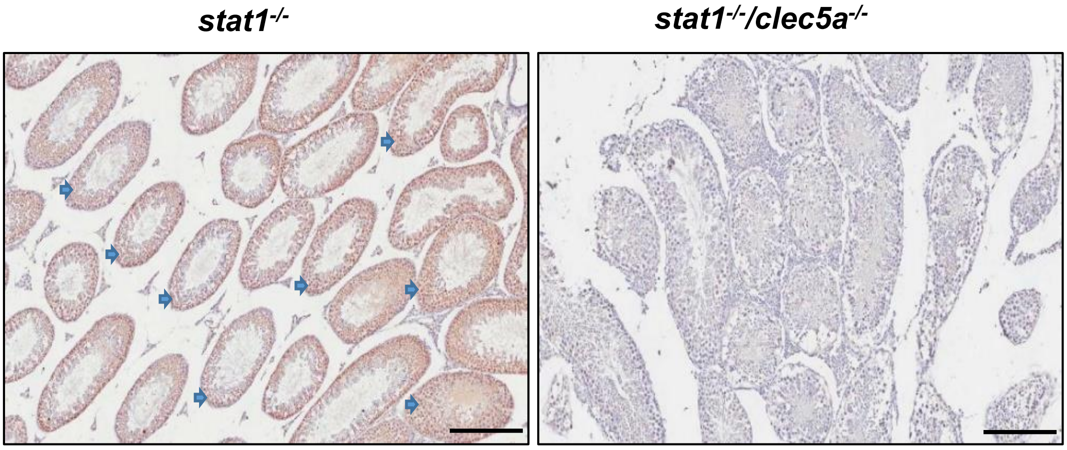

Supplementary Figure 3. CLEC5A is abundantly expressed in testicular tissues. Histological assay of testicular tissue sections from male *stat1*$^{-/-}$ and *stat1*$^{-/-}$*clec5a*$^{-/-}$ (double KO) mice without ZIKA infection. The tissue sections were stained with anti-CLEC5A mAbs (1 μg/ml) followed by a counterstain with hematoxylin (orange dotted area, blue arrows). Scale bar: 200 μm.

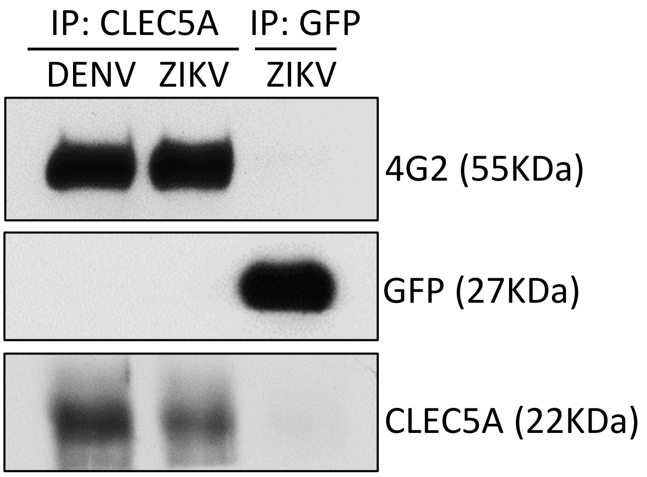

Supplementary Figure 4. ZIKV is capable of binding to CLEC5A. Western blot analysis of virus-CLECA protein complex detected via anti-4G2 (for virus), anti-GFP, or anti-CLEC5A from an immunoprecipitation of incubated DENV or ZIKV and CLEC5A. GFP is a negative control for virus binding.

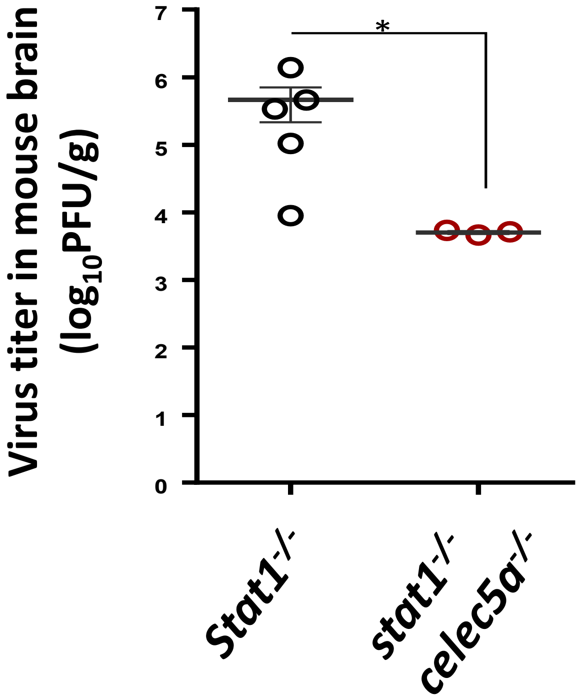

Supplementary Figure 5. ZIKA titer in brain tissues of mice. ZIKA titers from the brains of *stat1*$^{-/-}$ mice (n = 5) and *stat1*$^{-/-}$*clec5a*$^{-/-}$ mice (n = 3) at 7 dpi of ZIKA. The brain tissues were collected to determine virus titer by plaque-forming unit assay. The quantitative data was presented as mean ± SD. *$p < 0.05$.

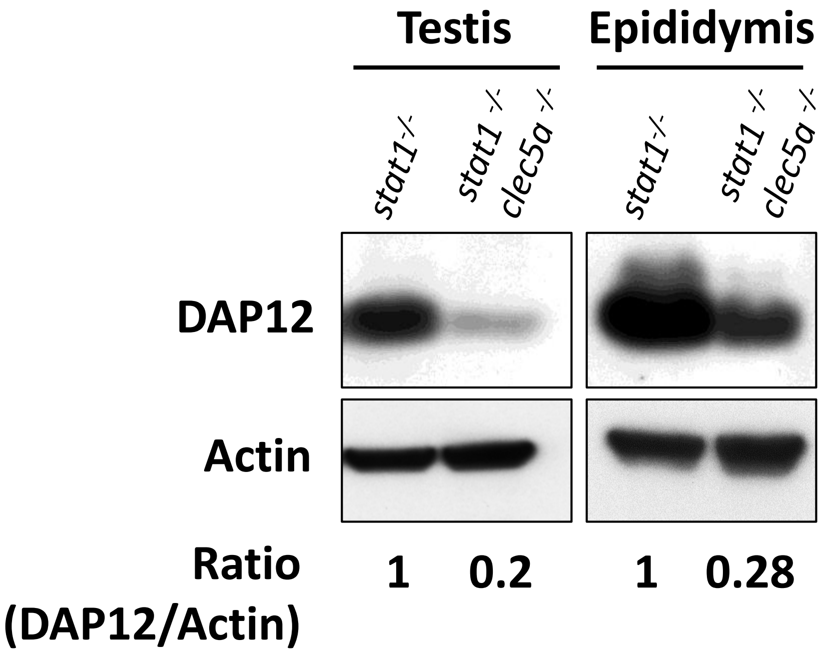

Supplementary Figure 6. CLEC5A deficiency causes decrease of testicular DAP12 expression. Western blot analysis of DAP12 protein expression in testis or epididymis of *stat*$^{-/-}$*clec5a*$^{-/-}$ mice compared to *stat*$^{-/-}$ mice.

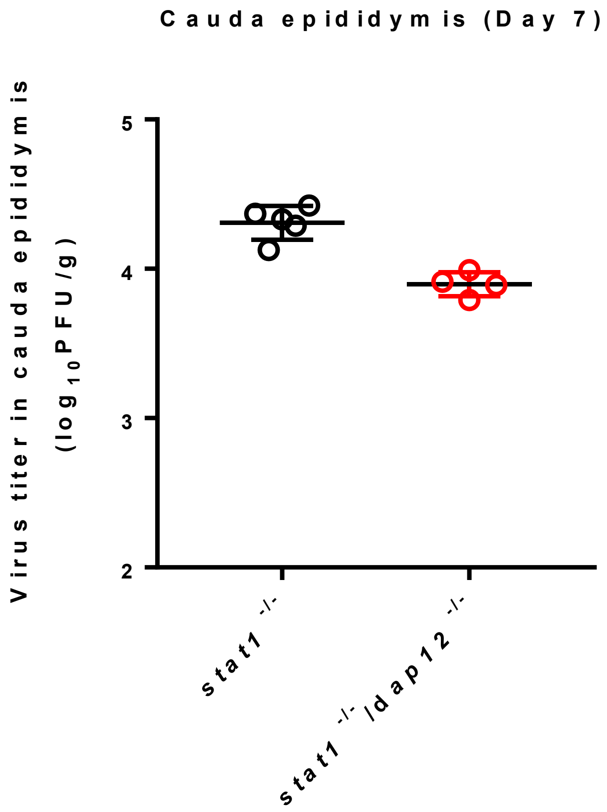

Supplementary Figure 7. ***Dap12*** **knockout reduces ZIKV titer.** Analysis of ZIKV titer in cauda epididymis tissue from ZIKV-infected *stat1*$^{-/-}$ and *stat1*$^{-/-}$*dap12*$^{-/-}$ mice (n ≥ 3) at 7 dpi. Quantitative data are presented as mean ± SD. *$p < 0.05$.

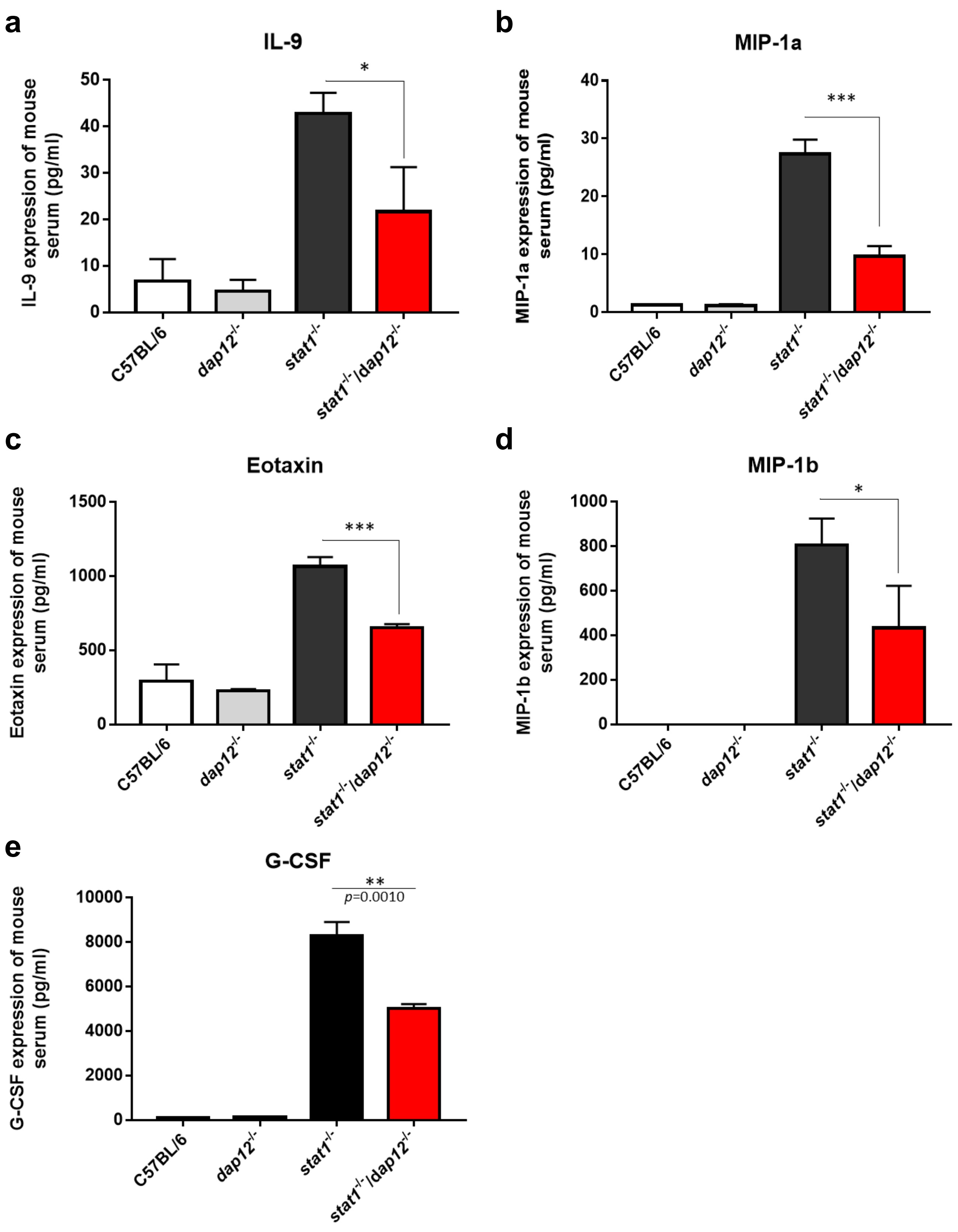

Supplementary Figure 8. Serum level of various cytokines in *dap12*$^{-/-}$ mice upon ZIKV infection. a-e Levels of serum cytokines IL-9 (**a**), MIP-1a (**b**), Eotaxin (**c**), MIP-1b (**d**), and G-CSF (**e**) in ZIKV-infected C57BL/6 or mutant mice were collected at 2 dpi and determined by a standard ELISA assay. Quantitative data are presented as mean ± SD. N.S., not significant. *$p < 0.05$. **$p < 0.01$. ***$p < 0.001$.

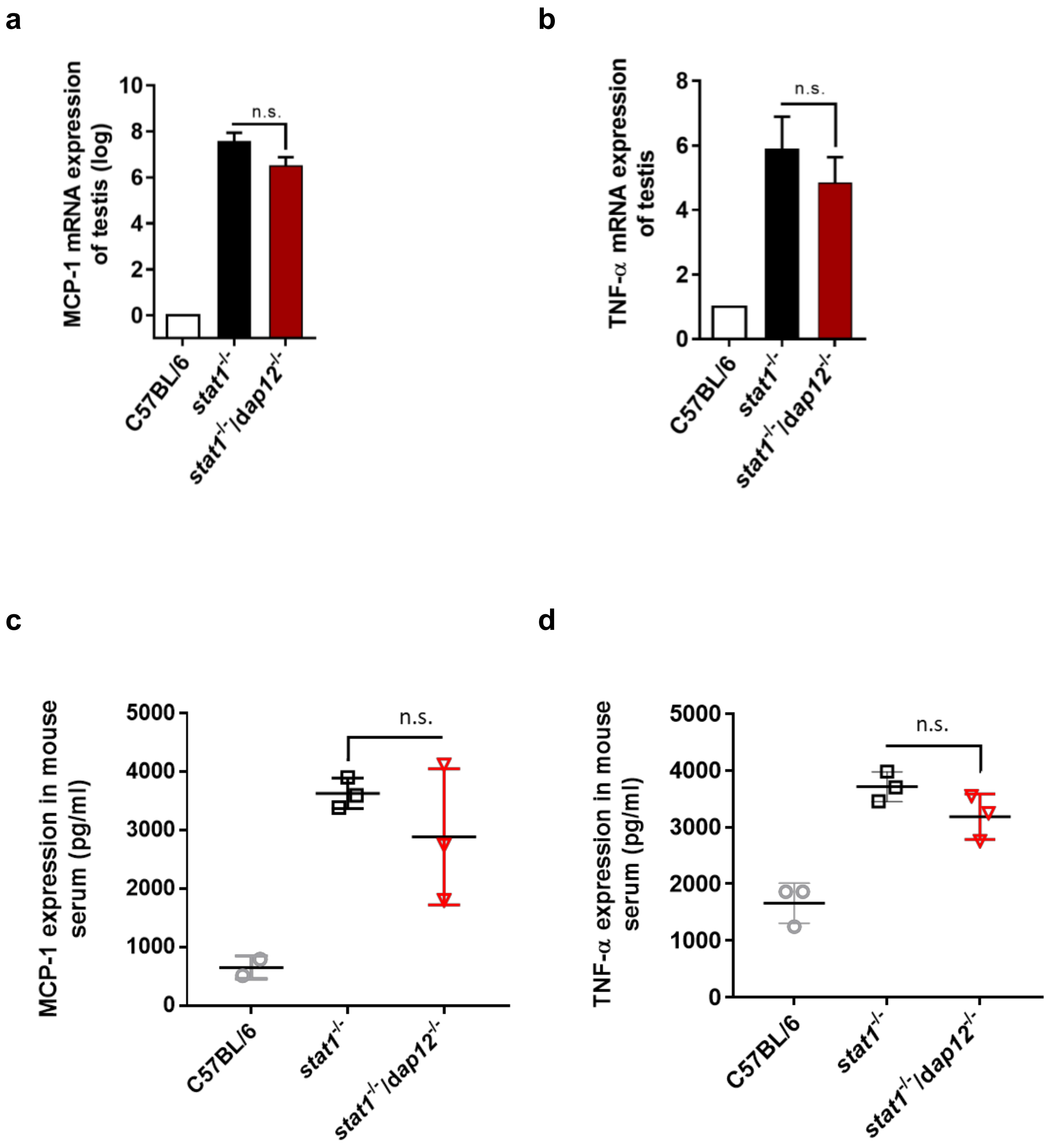

Supplementary Figure 9. Expression of MCP-1 and TNF-α in the serum and testes of ZIKV-infected *dap12*$^{-/-}$ mice or uninfected C57BL/6 mice. a-d Levels of chemokine MCP-1 and proinflammatory TNF-α cytokine in testes (**a**, **b**) and serum (**c**, **d**) from mutant or C57BL/6 mice with ZIKV infection. MCP-1 and TNF-α were detected from serum collected at 2 dpi via a standard ELISA assay, and transcripts were analyzed from testicular tissue harvested at 7 dpi via quantitative real-time PCR. Quantitative data are presented as mean ± SD. n.s., not significant.

a

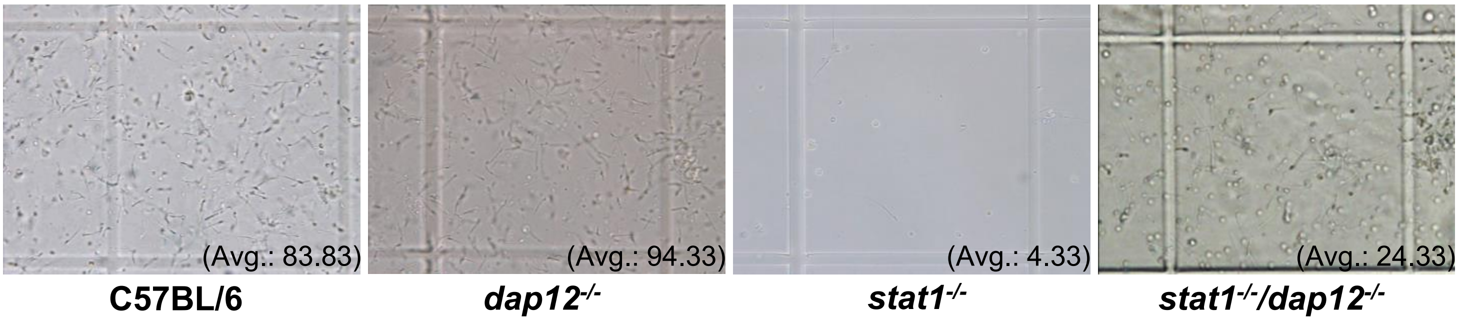

b

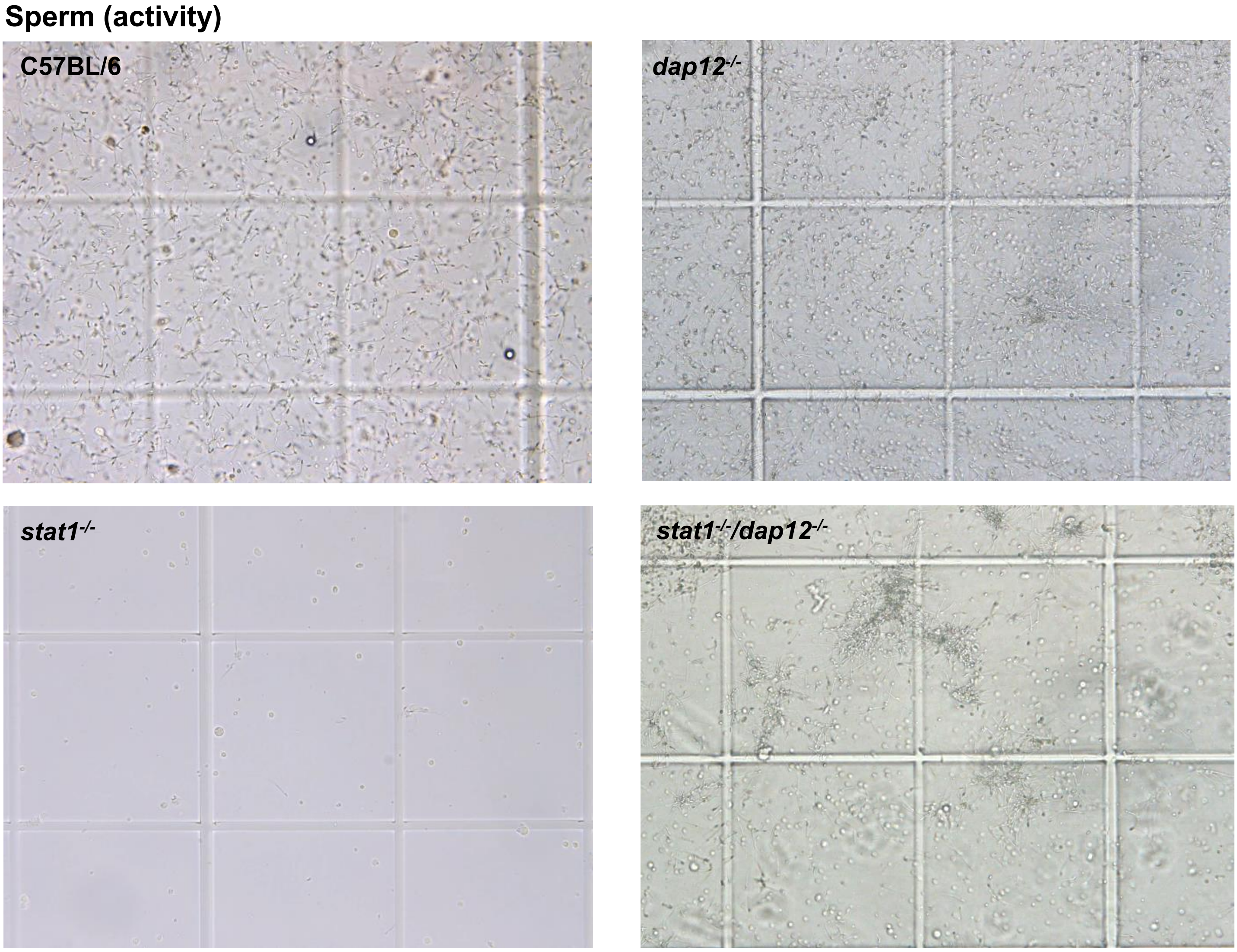

Supplementary Figure 10. Sperm motility and activity in mice upon ZIKV infection at 7 dpi. a The number of sperm in each ZIKA-infected mouse. **b** Video recordings of sperm motility and activity in male C57BL/6, *dap12*$^{-/-}$, *stat1*$^{-/-}$, and *stat1*$^{-/-}$*dap12*$^{-/-}$ mice infected with ZIKV. Mature sperm were isolated from the dissected cauda region of the epididymis, which was pre-incubated in Vitro Fert medium (K-RVFE 50, Cook Medical).

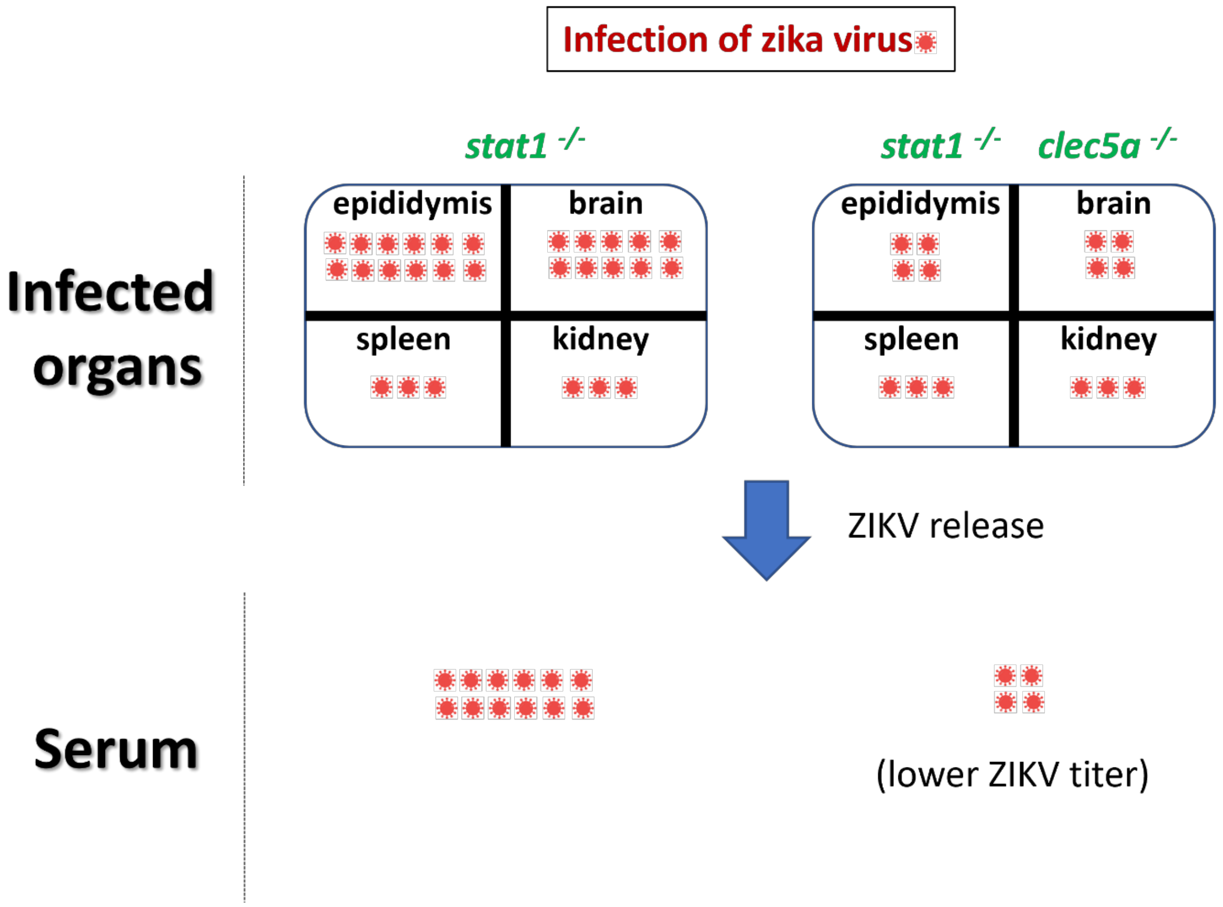

Supplementary Figure 11. Schematic illustration of how reduced ZIKV release from the epididymis and brain led to lower serum ZIKV. In WT (*stat*$^{-/-}$ *clec5a*$^{+/+}$) mice, ZIKV penetrates the blood-testis barrier (BTB)/blood-epididymis barrier (BEB) to infect Sertoli cells, the stromal cells of the testis, and activates CLEC5A+ macrophages. This activation induces testicular inflammation, thereby causing severe inflammatory reactions and damage to the BTB/BEB, which allows more ZIKV to invade the testis. A high titer of ZIKV can enter blood via the damaged BTB/BEB to cause viremia. In CLEC5A-deficient (*stat*$^{-/-}$*clec5a*$^{-/-}$) mice, *clec5a*$^{-/-}$ macrophages produce less proinflammatory cytokines, thereby causing less tissue damage and less ZIKV penetration to the testis. Because the BTB is relatively intact in *stat*$^{-/-}$*clec5a*$^{-/-}$ mice, less ZIKV is released to blood stream.
